# Laser balloon ablation of atrial fibrillation in a patient with a large common inferior trunk: a case report

**DOI:** 10.1093/ehjcr/ytae020

**Published:** 2024-01-08

**Authors:** Tomohiro Takiguchi, Masao Takemoto, Tokushi Koga, Takuya Tsuchihashi

**Affiliations:** Cardiovascular Centre, Steel Memorial Yawata Hospital, 1-1-1 Haruno-machi, Yahatahigashi-ku, Kitakyushu 805-8508, Japan; Cardiovascular Centre, Steel Memorial Yawata Hospital, 1-1-1 Haruno-machi, Yahatahigashi-ku, Kitakyushu 805-8508, Japan; Cardiovascular Centre, Steel Memorial Yawata Hospital, 1-1-1 Haruno-machi, Yahatahigashi-ku, Kitakyushu 805-8508, Japan; Cardiovascular Centre, Steel Memorial Yawata Hospital, 1-1-1 Haruno-machi, Yahatahigashi-ku, Kitakyushu 805-8508, Japan

**Keywords:** Atrial fibrillation, Case report, Catheter ablation, Common inferior trunk, Laser balloon

## Abstract

**Background:**

A balloon-based visually guided laser balloon (LB) ablation (LBA) is as effective and safe as radiofrequency ablation and cryoballoon ablation in curing patients with atrial fibrillation (AF). The third-generation LB is so compliant that it can be inflated to any pressure and size change of up to 41 mm with its maximal expansion, which enables maximum balloon/tissue contact regardless of the size or shape of each pulmonary vein (PV) ostium. A large common inferior trunk (CIT) with a structured, completely independent common ostium of both the right and the left inferior PVs completely conjoined prior to the junction with the left atrium is an extremely rare anatomical variant and an important triggering focus in paroxysmal AF.

**Case summary:**

We present a case of an LBA of AF in a patient with a large CIT of 34 mm in diameter. The laser energy was individually deployed to the right-sided and left-sided antra of the large CIT with the LB positioned at the ostium of the CIT’s right and left branches. The complete electrical isolation of the three PVs was achieved. The patient remained stable without any symptoms or AF recurrence 1 year post ablation.

**Discussion:**

The LBA, which is individually deployed to the right-sided and left-sided antra of the large CIT with the third-generation LB positioned at the ostium of the right and left branches of the CIT without laser energy deployment to the posterior wall of the CIT, may be one of the effective strategies for patients with large CITs.

**Learning points**

- The pre-procedural recognition of the venous anomalies such as large common inferior trunks (CITs) by three-dimensional imaging such as contrast-enhanced cardiac computed tomography and a careful planning of a strategy before the procedure are important for a smooth and safe ablation of atrial fibrillation.
- The laser balloon (LB) ablation, which is individually deployed to the right-sided and left-sided antra of the large CIT with the third-generation LB positioned at the ostium of the right and left branches of the CIT without laser energy deployment to the posterior wall of the CIT, may be one of the effective strategies for patients with large CITs.

## Introduction

The balloon-based visually guided laser balloon (LB) ablation (LBA) is as effective and safe as radiofrequency ablation and cryoballoon (CB) ablation in treating patients with atrial fibrillation (AF)^1–3^ even with a left common pulmonary vein (PV).^4^ A common inferior trunk (CIT) of the PVs is a comparably rare anatomical variant and an important triggering focus in paroxysmal AF.^5^

## Summary figure

**Figure:**
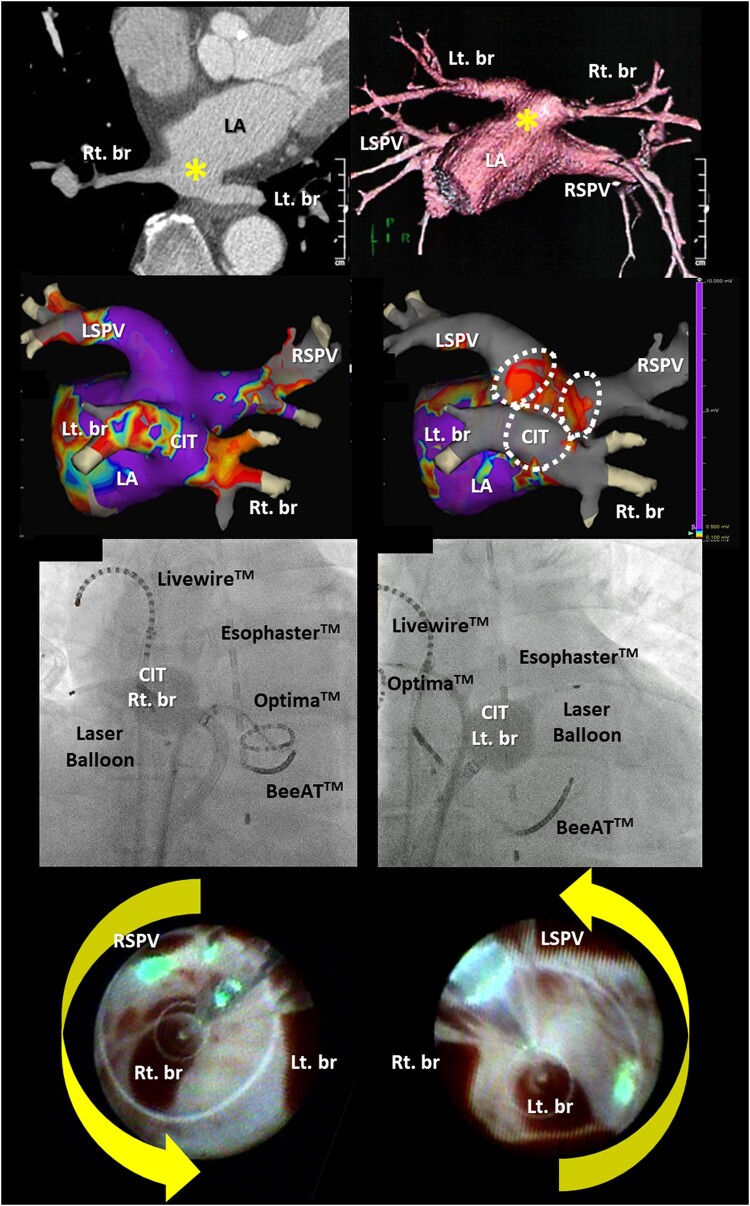


## Case presentation

**Figure 1:**
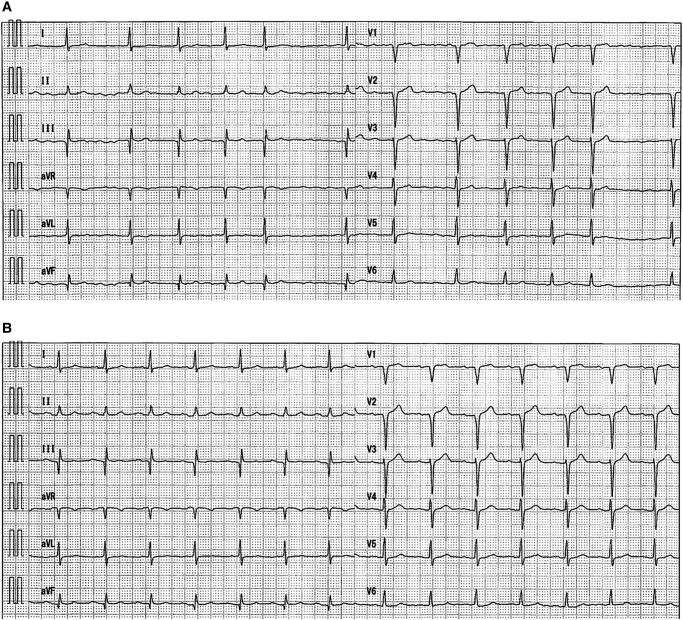
Twelve-lead electrocardiograms during atrial fibrillation (*A*) and 1 year post ablation (*B*).

**Figure 2:**
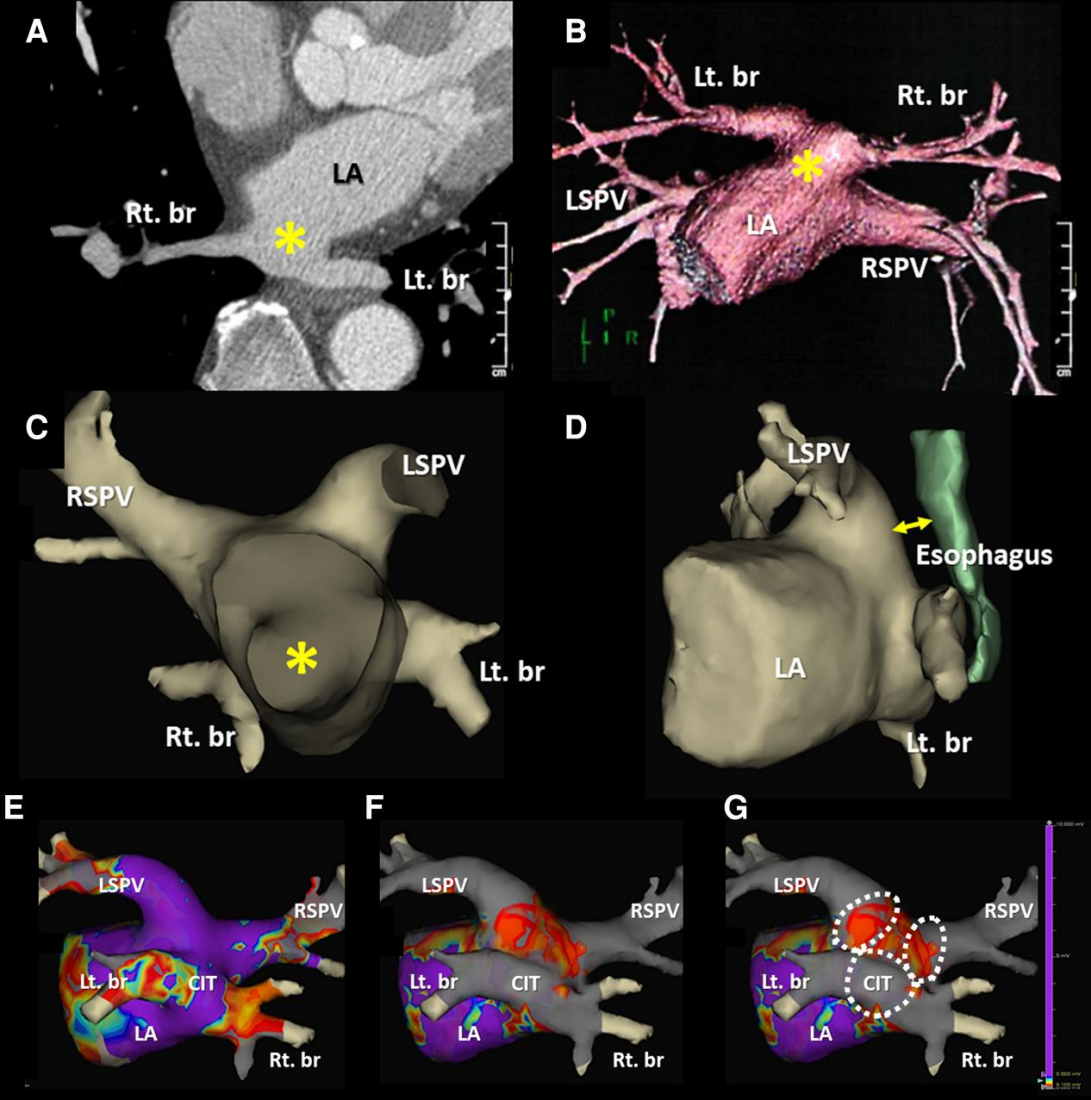
A computed tomography projection in the oblique axial view presents the inferior pulmonary veins entering the left atrium via a common ostium (asterisk in *A*). A volume-rendered three-dimensional computed tomography image of the common ostium of the right and left inferior pulmonary veins (*B*) and the endoscopic view from the front (*C*) and the left (*D*) demonstrate the presence of a large common inferior trunk of the pulmonary veins arising from the posterior wall of the left atrium. The EnSite™ images viewed from the back before (*E*) and after (*F* and *G*) ablation using a third-generation (X3) laser balloon for the three pulmonary veins. The white dotted lines represent the ablation lines of the X3-LB, which were deduced from the findings seen in *Figure 3E–H*. LSPV, left superior pulmonary vein; RSPV, right superior pulmonary vein; Lt. br, left branch; Rt. br, right branch.

A 73-year-old male patient with a history of hypertension, diabetes mellitus, heart failure, dyslipidaemia, and a cerebral infarction was admitted to our hospital to undergo an ablation of paroxysmal AF (*Figure 1A*). He was receiving optimized medical therapy for his conditions. On admission, he had a blood pressure of 130/68 mmHg and regular heart rate of 72 beats/min. A precordial auscultation revealed normal cardiac and respiratory sounds. A 12-lead electrocardiogram revealed a sinus rhythm. An echocardiography revealed a normal left ventricular ejection fraction and left atrial (LA) enlargement of 42.8 mm. The patient’s CHADS_2_/CHA_2_DS_2_-VASc score was 5/7. Prior to the procedure, a contrast-enhanced cardiac computed tomography (CT) revealed an atypical PV, wherein the right and left superior PVs had normal separate ostia, while the two right and left inferior PVs had a common ostium, exhibiting a CIT^6,7^ (*Figure 2A–C*). Because the diameters of the antrum of the right and left superior PVs and the junction between the CIT and the LA were 34, 35, and 34 mm, respectively, we decided to perform ablation using an LB, which has a maximum diameter of 41 mm.

**Figure 3:**
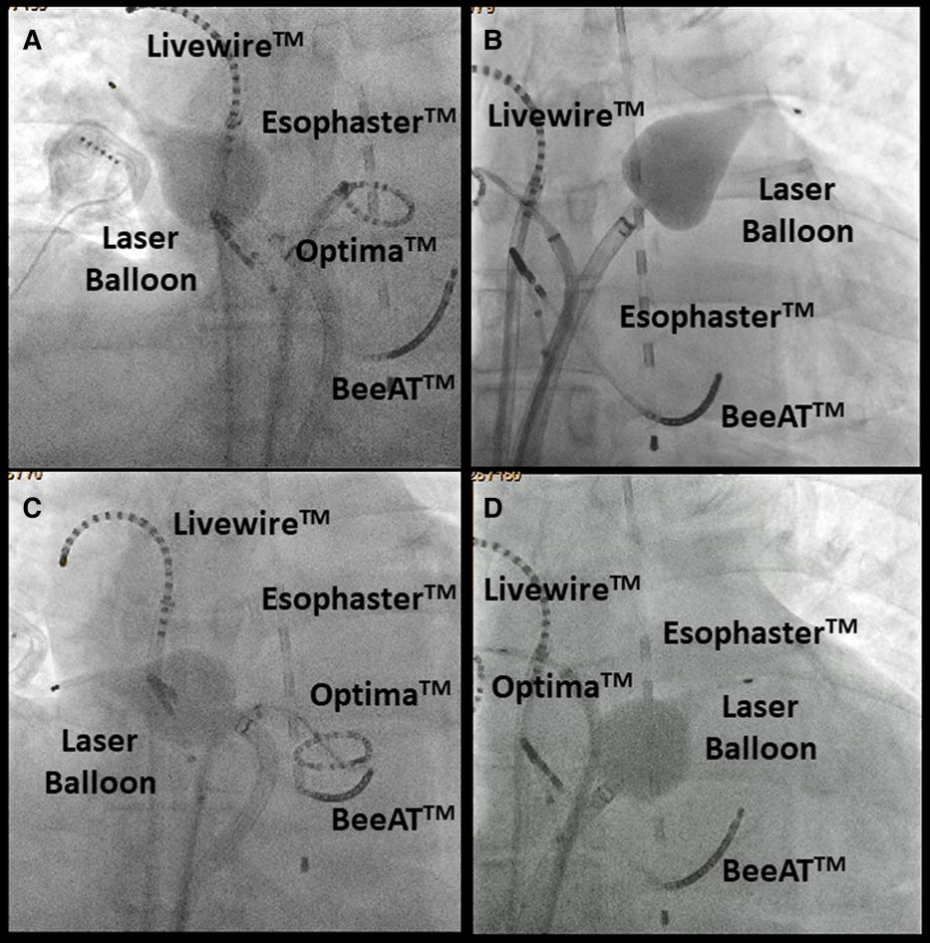

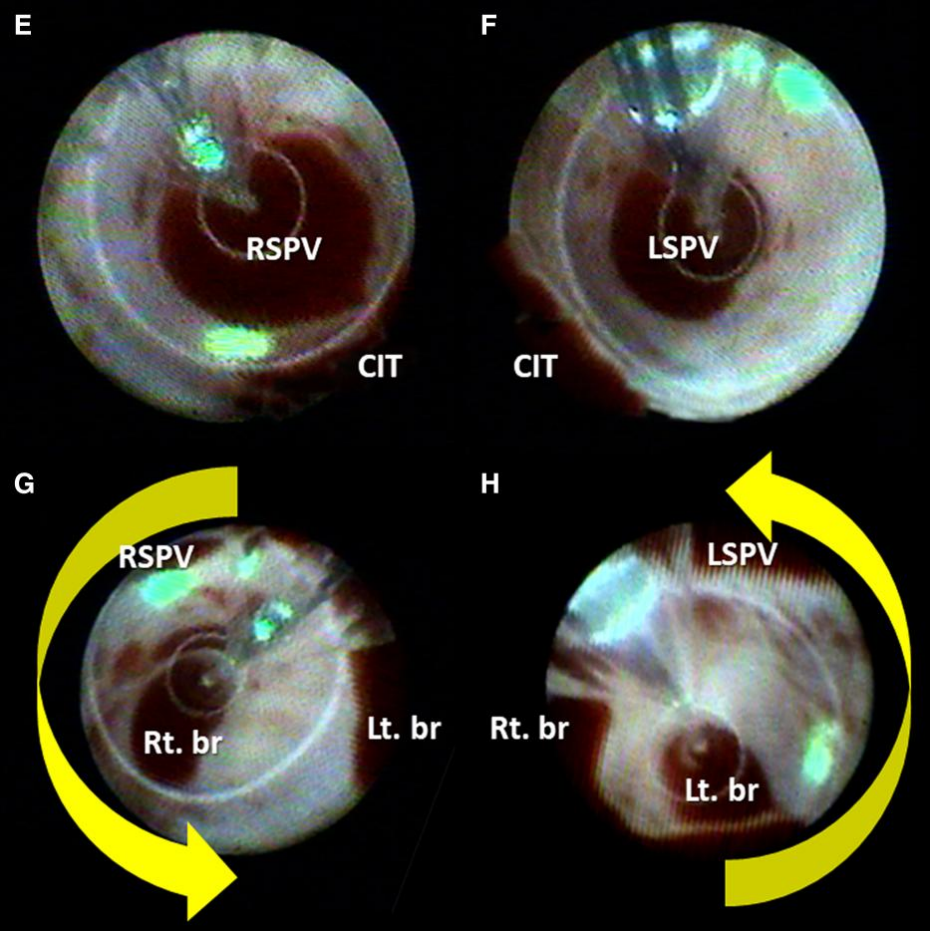
The fluoroscopic images demonstrate that the third-generation (X3) laser balloon (X3-LB) was positioned at the ostium of the right (*A*) and left (*B*) superior pulmonary veins and the right (*C*) and left (*D*) branches of the common inferior trunk. The endoscopic views of the right (*E*) and left (*F*) superior pulmonary veins and the common inferior trunk with the X3-LB positioned at the ostium of the right (*G*) and left (*H*) branches of the common inferior trunk. The arrows demonstrate the line of the laser energy administration. LSPV, left superior pulmonary vein; RSPV, right superior pulmonary vein; Lt. br, left branch; Rt. br, right branch.

A temperature probe (Esophaster™, Japan Lifeline) for monitoring the oesophageal temperature was inserted and placed between the levels of the left superior PV and the CIT, and a deflectable catheter, a BeeAT™ (Japan Lifeline), was placed in the coronary sinus and right atrium (*Figure 3A–D*). Following a double transseptal puncture, a steerable 12 Fr transseptal sheath (HeartLight Deflectable Sheath™, CardioFocus) and an 8.5 Fr sheath (Biosense Webster) were placed into the LA. The LA and three PVs were reconstructed using a three-dimensional (3D) mapping system (EnSite™, Abbott) with a circular mapping catheter (Optima™, Abbott) and the LA was confirmed to have an almost normal voltage (*Figure 2E*). The third-generation (X3) LB (HeartLight X3 Catheter™, CardioFocus) was navigated to the target right (*Figure 3A*) and left (*Figure 3B*) superior PVs using a 12F sheath and inflated for an optimal PV occlusion at an antral balloon position.^8^ Laser energy was deployed individually, with a 30–50% overlap in the manual energy mode. The rapid mode is continuously delivered and is not defined by this overlap percentage (*Figure 3E* and *F*). Next, the X3-LB was positioned at the ostium of the right branch of the CIT (*Figure 3C*) and inflated for optimal PV occlusion at the antral balloon position. The endoscopic view of the right branch of the CIT revealed that the right side of the LB came into complete contact with the right-sided antrum of the CIT, but the left side of the LB was in contact with the posterior wall of the CIT (*Figure 3G*). Conversely, the X3-LB was positioned at the ostium of the left branch of the CIT (*Figure 3D*) and inflated for optimal PV occlusion at the antral balloon position. The endoscopic view of the left branch of the CIT revealed that the left side of the LB came into complete contact with the left-sided antrum of the CIT, but the right side of the LB was in contact with the posterior wall of the CIT (*Figure 3H*). Thus, the laser energy was then individually deployed to the right-sided (*Figure 3G*) and left-sided (*Figure 3H*) antra of the CIT with the X3-LB positioned at the ostium of the CIT’s right (*Figure 3C*) and left (*Figure 3D*) branches. During ablation of the right superior PV and CIT, continuous right phrenic nerve pacing in the superior vena cava and monitoring of compound motor action potentials were performed to prevent phrenic nerve injury.^8^ Finally, the complete extensive electrical isolation of the three PVs, including that of the right-sided and left-sided PV carinas, was achieved without any complications (*Figure 2F* and *G*). The patient remained stable without any symptoms or AF recurrence 1 year post ablation (*Figure 1B*).

## Discussion

A large CIT with a structured, completely independent common ostium of both the right and the left inferior PVs completely conjoined prior to the junction with the LA (*Figure 2A* and *B*), such as in the present case, is an extremely rare anatomical variant, and the rate of prevalence is <1% in patients with AF.^5^

Based on the characteristic dissection seen in this case, we discussed how the ablation lesions were designed pre-operatively. It has been reported that the non-isolation of the PV carinas after a successful PV antrum isolation may be an independent predictor of AF recurrence.^9^ Moreover, recent reports have demonstrated that an extensive ‘tri-circle’ ablation^5,6^ of the three PVs might be an effective strategy in patients with a CIT. Therefore, we pre-operatively planned to perform the ‘tri-circle’ ablation^5,6^ technique of the right and left superior PVs and CIT including the right-sided and left-sided PV carinas in our patient. The CB ablation of a CIT is a useful therapeutic tool for treating AF.^10^ However, the CB was not suitable for our patient because the CB diameter (measuring 28 mm) was smaller than that of the antra of the right and left superior PVs and the CIT, which were 34, 35, and 34 mm, respectively. Further, the branches of the CIT were not coaxial from the transseptal site of the right atrium (*Figure 2A*). This may have caused poor contact of the CB with the tissue, affecting cryothermal energy delivery. Additionally, a recent report demonstrated that it might be difficult to stabilize the radiofrequency catheter on the ridge between the two inferior PVs, particularly on the posterior wall in patients with CITs.^11^ Conversely, the X3-LB is so compliant that it can be inflated to any pressure and can change size by up to 41 mm with maximal expansion, which enables maximum balloon/tissue contact regardless of the size or shape of each PV antrum. This contributes to an extensive stable ablation of both ipsilateral PVs^4^ including the carinas. However, physicians should pay attention to the decreasing compliance of the LB with an increasing balloon pressure, which may make the occlusion more difficult.^4^ In fact, occlusion of blood flow is necessary to deliver energy to the tissue and avoid char due to energy delivery from the optical laser. Thus, LBA was thought to be suitable for this particular patient instead of a radiofrequency ablation catheter. Finally, we decided to perform an extensive ‘tri-circle’ ablation using an LB with extreme caution, as it is a challenging strategy. Interestingly and fortunately, even though there was a non-coaxial placement of the LB in the large CIT (*Figure 2A*), the LB was able to completely occlude the blood flow, and the laser energy was individually deployed to the right-sided (*Figure 3G*) and left-sided (*Figure 3H*) antra of the CIT in this case. The X3-LB was positioned at the ostium of the CIT’s right (*Figure 3C*) and left (*Figure 3D*) branches without laser energy deployment to the posterior wall of the CIT as this could contribute to the development of PV stenosis. Finally, we were able to successfully achieve an extensive ‘tri-circle’ ablation including the CIT with a bilateral laser energy delivery (*Figure 2F* and *G*). In view of these findings, this ablation strategy including both the right-sided and the left-sided PV carinas by LBA may be a more suitable method than CB or radiofrequency catheter ablation in patients with a large CIT of the PVs. However, because the anatomy of the CITs (such as the ostium dimension and neck length) may vary, a pre-procedural recognition of these venous anomalies by 3D imaging such as a contrast-enhanced cardiac CT and careful planning of a strategy before the procedure is important for a smooth and safe ablation of AF.

When performing ablation in patients with a CIT, if the oesophagus runs along the CIT and is in contact with the posterior wall of the LA and CIT, the procedure may be technically difficult to perform because LBA has to cross directly over the oesophagus twice. This could be a disadvantage of this procedure. Thus, we might not suggest an LBA for these patients because of an increased risk of an atrio-oesophageal fistula.^12^ The recent advancements in technologies, including pulsed-field ablation, may allow for a selective ablation of atrial myocytes, offering an ablation method that is significantly safer for these patients in contrast to thermal ablation modalities.^12^ In the present case, fortunately, the patient’s oesophagus ran along the left side of the CIT and came in contact only with the left bottom area of the CIT and was 8 mm away from the posterior wall of the LA (*Figure 2D*). Finally, the AF was successfully treated by using LBA without any complications.

## Conclusion

Laser balloon ablation, individually deployed to the right-sided and left-sided antra of a large CIT with the X3-LB positioned at the ostium of the right and left branches of the CIT without laser energy deployment to the posterior wall of the CIT, may be an effective strategy for AF ablation in patients with large CITs.

## Data Availability

The data underlying this article will be shared on reasonable request to the corresponding author.

## Lead author biography

Dr Tomohiro Takiguchi received his MD degree from Kyushu University (Fukuoka, Japan) in 2011. Since 2021, he has been working as a cardiologist at the Social Medical Corporation Steel Memorial Yawata Hospital. His area of medical interest is heart failure, arrhythmias, and coronary artery disease.
